# The mouse alpha-globin cluster: a paradigm for studying genome regulation and organization

**DOI:** 10.1016/j.gde.2020.10.003

**Published:** 2021-04

**Authors:** A Marieke Oudelaar, Robert A Beagrie, Mira T Kassouf, Douglas R Higgs

**Affiliations:** 1 Max Planck Institute for Biophysical Chemistry, Göttingen, Germany; 2 Laboratory of Gene Regulation, MRC Weatherall Institute of Molecular Medicine, Radcliffe Department of Medicine, University of Oxford, Oxford, UK

## Abstract

The mammalian globin gene clusters provide a paradigm for studying the relationship between genome structure and function. As blood stem cells undergo lineage specification and differentiation to form red blood cells, the chromatin structure and expression of the α-globin cluster change. The gradual activation of the α-globin genes in well-defined cell populations has enabled investigation of the structural and functional roles of its enhancers, promoters and boundary elements. Recent studies of gene regulatory processes involving these elements at the mouse α-globin cluster have brought new insights into the general principles underlying the three-dimensional structure of the genome and its relationship to gene expression throughout time.

**Current Opinion in Genetics and Development** 2021, **67**:18–24

This review comes from a themed issue on **Genome architecture and expression**

Edited by **Susan M Gasser** and **Gerd A Blobel**

For a complete overview see the Issue and the Editorial

Available online 19th November 2020

**https://doi.org/10.1016/j.gde.2020.10.003**

0959-437X/© 2020 The Authors. Published by Elsevier Ltd. This is an open access article under the CC BY license (http://creativecommons.org/licenses/by/4.0/).

## Introduction

Since the DNA sequences of mammalian genomes have been established, a major goal has been to understand how this linear code is deciphered to produce complex multicellular organisms. A key component of this process is the selection and maintenance of gene expression programs by proteins and nucleic acids that recognize the linear sequence of the regulatory elements in the DNA and thereby regulate gene expression. However, reading the DNA code is complicated by its epigenetic profile: how it is packaged into chromatin and how the associated DNA and histone proteins are covalently modified. In addition, gene regulation is related to the organization of chromatin into topologically associating domains (TADs and smaller subTADs), which broadly correspond to regions of the genome containing enhancers and their cognate promoters, flanked by convergent boundary elements [1,2].

Here we review how comprehensive analyses of the fundamental regulatory elements within a small TAD containing the mouse α-globin cluster have contributed to our understanding of the general principles underlying gene regulation and genome organization and their relationship to each other. An important advantage of studying the α-globin cluster is that these genes are specifically activated in erythroid cells, which are very well characterized and can be purified at sequential stages of erythroid differentiation. This has allowed for precise determination of the order of regulatory events as the α-globin genes are switched on during erythropoiesis. Importantly, and in contrast to many other gene loci used to study the principles of gene regulation, the cell fate decisions underpinning erythropoiesis are largely unaffected by changes in α-globin gene expression [3,4]. This means that it is possible to perturb the structure and function of the regulatory elements of the α-globin cluster without changing the cell lineage, its differentiation or maturation, thereby allowing direct interpretation of experimental variations in the *cis*-acting regulatory landscape without confounding changes in the *trans*-acting environment.

## Mouse erythropoiesis

**Figure 1:**
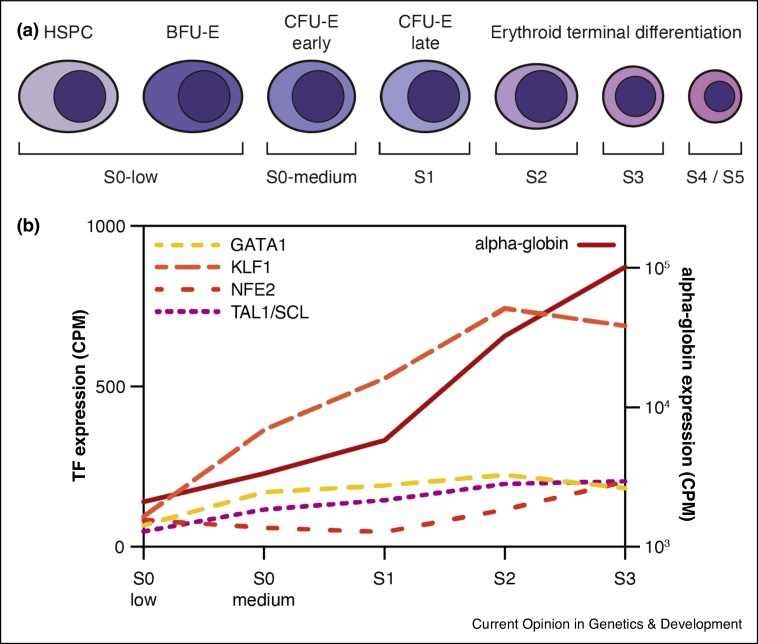
Expression of α-globin and key erythroid transcription factors during erythropoiesis. **(a)** Using flow cytometry, sequential stages of *in vivo* erythroid differentiation can be isolated from mouse fetal livers. The S0-low population contains early erythroid progenitors, predominantly Burst-Forming Unit-Erythroid (BFU-E) cells; the S0-medium population contains primarily early Colony-Forming Unit-Erythroid (CFU-E) cells; the S1 population contains the last CFU-E cell division before terminal differentiation; the S2–S5 populations contain progressively more mature terminally differentiating erythroblasts. **(b)** Expression in counts per million (CPM) of erythroid transcription factors (TF) (left Y-axis) and α-globin (right Y-axis) throughout erythropoiesis as determined by single-cell RNA-seq [9^••^].

Definitive erythropoiesis in mice occurs via a continuous process of lineage specification and differentiation from hematopoietic stem cells (HSCs), via multipotent progenitors, early erythroid progenitors, and fully committed erythroid progenitors [5,6]. Pure cell populations from each of these stages can be isolated from fetal liver using flow cytometry [7,8^••^]. The transition from early erythroid progenitors to terminally differentiated erythroid cells in mouse has been divided into six sequential phases (subsets S0, S1, S2, S3, S4 and S5) [7] (Figure 1a). The point of commitment to terminal differentiation is synchronized with the cell cycle clock: erythroid progenitors are synchronized in S-phase and undergo a rapid cell cycle which appears to act as a checkpoint for all subsequent changes in chromatin and gene expression [7,8^••^]. Expression of α-globin and key erythroid transcription factors involved in its regulation are switched on at this transition and further upregulated during subsequent differentiation [8^••^,9^••^] (Figure 1b).

## The regulatory elements of the α-globin cluster

**Figure 2:**
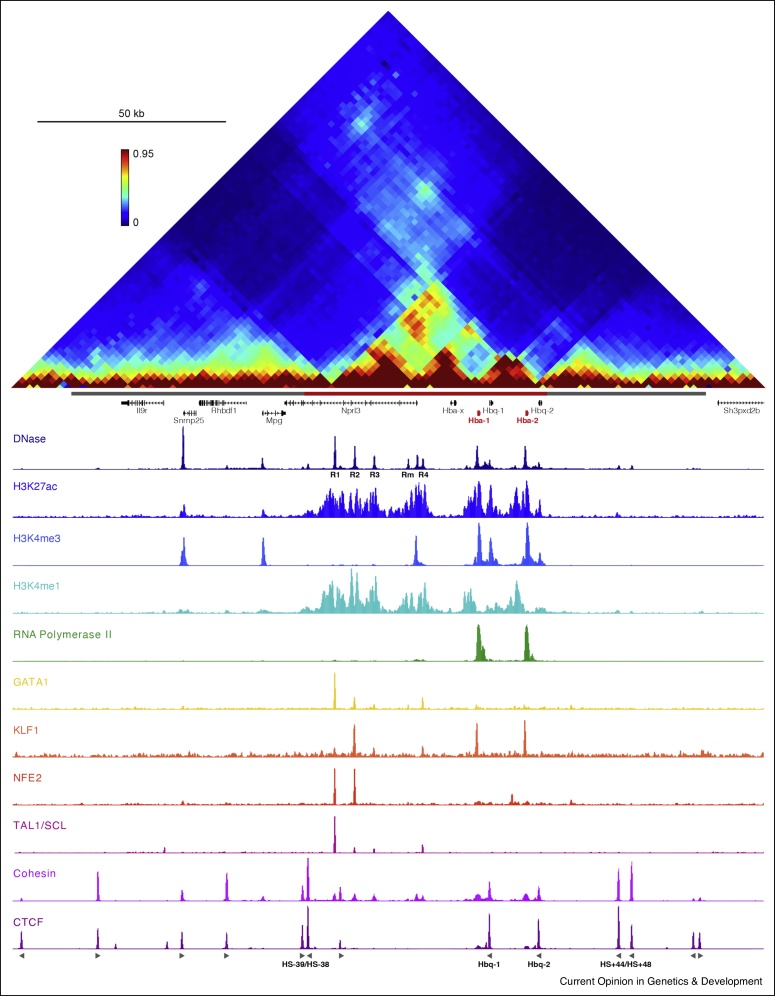
The regulatory elements and higher-order structure of the mouse α-globin locus in mature erythroid cells. From top to bottom: 3C contact matrix of 200 kb spanning the mouse α-globin cluster (Tiled-C); accessible chromatin (DNase-seq); ChIP-seq of histone modifications (H3K27ac, H3K4me3 and H3K4me1); occupancy of RNA Polymerase II, erythroid transcription factors (GATA1, KLF1, NFE2 and TAL1/SCL), the cohesin complex (RAD21) and CTCF. The grey and red bars below the matrix represent the TAD (chr11:32 080 000–32 245 000) and subTAD (chr11:32 136 000–32 202 000), respectively. The α-globin genes are highlighted in red and its enhancers are indicated below the DNaseI profile. The orientation of CTCF motifs are shown below the CTCF profile and CTCF-binding elements of interest are indicated. Coordinates (mm9): 32 060 000–32 260 000.

The mouse α-globin cluster is located in a ∼65 kb erythroid-specific subTAD, which is contained within a larger ∼165 kb TAD present in all tested cell types [9^••^] (Figure 2). The locus includes the embryonic ζ-globin gene (*Hba-x*) and a pair of almost identical adult α-globin genes (*Hba-1* and *Hba-2*). The cluster also contains two θ-globin genes (*Hbq-1* and *Hbq-2*) of unknown function. All of these genes are regulated by a set of five erythroid-specific enhancer elements (R1, R2, R3, Rm and R4) upstream of the cluster. Four of these enhancers (R1–R3 and Rm) are located in introns of the housekeeping gene *Nprl3*. The enhancer cluster fulfils the definition of a superenhancer [10]. It has been suggested that the constituent elements of superenhancers might function synergistically. However, careful genetic dissection of the cluster by deleting enhancers individually and in informative combinations has thus-far revealed only additive interactions between the α-globin enhancers [4]. The α-globin genes and their enhancers are flanked by multiple largely convergent CCCTC-binding factor (CTCF)-binding elements at the boundaries of the subTAD [11]. With exception of the mouse-specific Rm element, the *Hbq-2* gene and some CTCF-binding sites, the mouse elements are conserved in the human α-globin locus [12].

The enhancers and promoters in the α-globin locus contain binding sites for key erythroid-specific and general transcription factors (Figure 2). At least some of the erythroid-specific transcription factors bind the enhancers of the α-globin cluster and initiate changes in chromatin modifications long before the globin genes are upregulated [13], which likely serves to prime these elements for activation. In contrast to the erythroid-specific transcription factor occupancy of the enhancers and promoters in the α-globin cluster, the boundary elements recruit CTCF in all cell types tested [11].

Overall, the α-globin subTAD is typical of many other developmental gene loci in the genome and therefore offers an excellent model to establish the general principles underlying the relationship between genome structure and function mediated via the fundamental regulatory elements.

## The large-scale organization of the α-globin cluster

TADs and subTADs are defined as domains in which DNA sequences interact more frequently with other DNA sequences within the domain compared to those outside the domain [14,15]. It has been proposed that TAD structures are formed by a dynamic process of loop extrusion, in which the cohesin complex translocates along the chromatin and brings all sequences within a domain into close proximity at some point during this process [16,17]. The extruded domains are delimited by CTCF-binding boundary elements, which have been shown to stall the translocation of cohesin and stabilize the stalled complex on chromatin. Importantly, the ability to stabilize cohesin is mediated by the N-terminal region of CTCF and is therefore determined by the orientation of the CTCF-binding sites [18, 19, 20].

The structure of the subTAD containing the α-globin cluster is consistent with this model (Figure 2). This domain is flanked by largely convergent CTCF-binding elements, which co-localize with cohesin [11]. Based on analysis by Chromosome Conformation Capture (3C) [9^••^], super-resolution imaging [21] and polymer physics models [22], it has been shown that the α-globin subTAD only forms a prominent structure in erythroid cells. This suggests that the process of loop extrusion is enhanced during erythroid differentiation. Interestingly, CTCF-independent cohesin peaks appear at the enhancers and promoters of the α-globin cluster in erythroid cells [11]. It is possible that loading or stalling of cohesin at these elements is specifically increased in erythroid cells, where these elements are active and bound by transcription factors and co-factors.

## Interactions between the regulatory elements of the α-globin cluster

The long-standing question of how specific communication between enhancers and promoters is mediated may be answered – at least in part – by the loop extrusion model. Since loop extrusion predicts that all sequences within a (sub)TAD interact at some point, loop extrusion could bring enhancers and promoters within a shared domain into close proximity. Subsequent interactions between chromatin, intermediary proteins and RNA may reinforce or stabilize such interactions, possibly by the formation of non-membrane bound nuclear compartments enriched in activating molecules [23,24].

3C experiments at the α-globin cluster have demonstrated that the enhancers and promoters come into close physical proximity in erythroid cells [25]. Furthermore, multi-way 3C experiments have identified complex structures in which multiple enhancer elements simultaneously interact with the promoters in a regulatory hub [26^•^], consistent with the existence of non-membrane bound nuclear compartments. Based on the loop extrusion model, it is expected that the boundary elements delimit the interactions between the α-globin enhancers and the genes located outside of the subTAD. Indeed, deletion of the boundary elements upstream of the enhancers (HS-38 and HS-39) creates an extension of the subTAD, resulting in ectopic enhancer-promoter interactions and strong upregulation of the upstream genes *Rhbdf1* and *Mpg* [11,27]. However, deletion of the downstream CTCF-binding elements (HS + 44, HS + 48, *Hbq-1* and *Hbq-2*) has no effect on the expression of the downstream genes [28]. Instead, the active *Hba-2* promoter appears to act as the downstream boundary of the subTAD, consistent with the observation that some TAD borders overlap with active promoters [15]. Interestingly, chemical inhibition of transcription does not change the 3D conformation of the locus, which suggests that the boundary function of the promoter is not dependent on active transcription *per se* [21].

## The regulation of α-globin activation during erythropoiesis

To obtain a complete understanding of gene regulation during differentiation and development, it is important to characterize the order of events that culminate in efficient transcription of developmental gene loci. Mouse erythropoiesis provides an unparalleled platform for the temporal dissection of gene regulatory events, as pure populations representing sequential stages of differentiating erythroid progenitors can be readily purified using flow cytometry [7,8^••^] (Figure 1).

**Figure 3:**
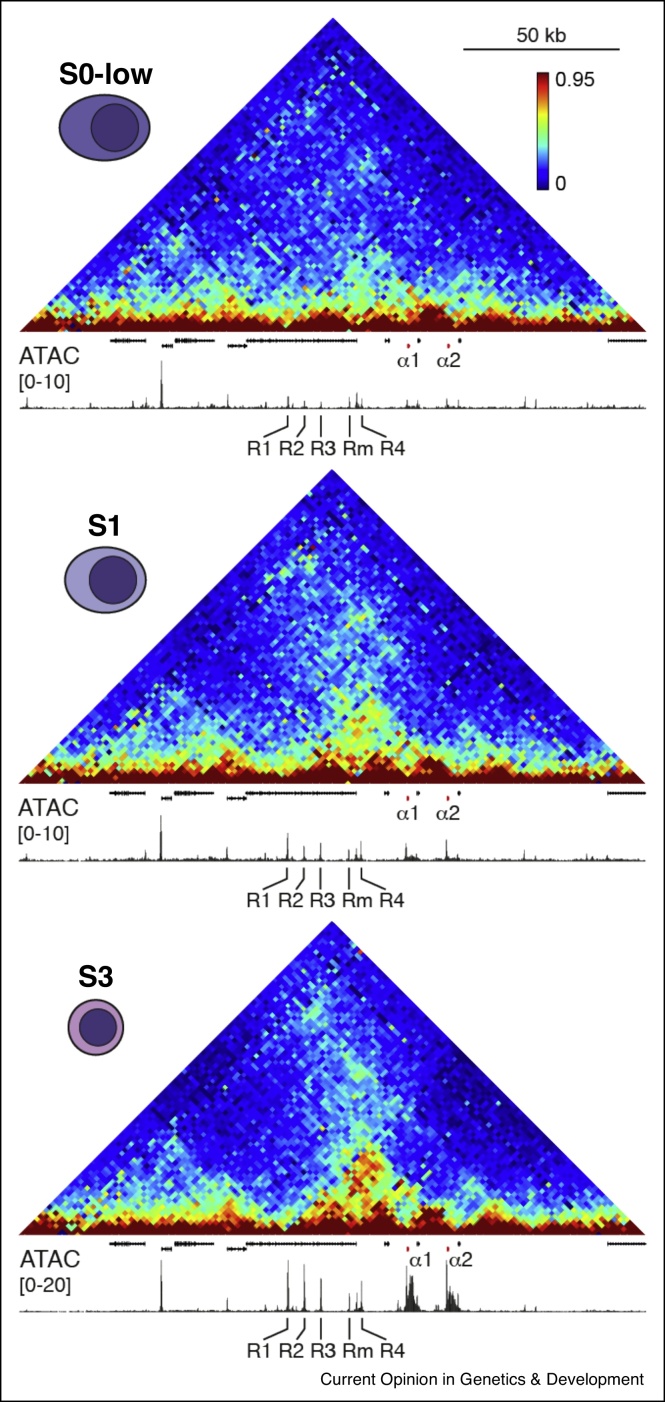
Activation of the α-globin cluster during erythropoiesis. The order of events during activation of the α-globin cluster has been determined based on integrated analysis of gene expression, chromatin accessibility and chromatin structure throughout *in vivo* erythropoiesis. The figure displays 3C contact matrices (Tiled-C) and open chromatin (ATAC) profiles in a subset of stages of erythroid differentiation [9^••^]. This clearly shows that the enhancers of the α-globin cluster become accessible within a pre-existing TAD before α-globin activation. This is followed by chromatin re-organization and the formation of an erythroid-specific subTAD in which the α-globin enhancers and promoters form specific interactions. Through differentiation, accessibility and interactions between the enhancers and promoters gradually increase, concomitant with progressive upregulation of gene expression.

At the mouse α-globin cluster, the appearance of regions of open chromatin, binding of key transcription factors and changes in the structure of the TAD and subTAD have been documented throughout erythropoiesis [9^••^,^13^] (Figure 3). The TAD containing the α-globin cluster is present in pluripotent embryonic stem cells, multipotent HSCs, and other early hematopoietic progenitors [9^••^], consistent with the ubiquitous CTCF occupancy of the boundary elements of the TAD [11]. The first steps in α-globin gene activation occur in very early erythroid progenitors and involve the α-globin enhancers becoming accessible [9^••^] and recruiting key erythroid transcription factors [13]. The formation of open chromatin at the enhancers occurs before changes in chromatin organization and activation of α-globin RNA expression. The subsequent chromatin reorganization involves the appearance of a smaller self-interacting domain (subTAD) within the larger TAD, in which interactions between enhancers and promoters are formed. These specific enhancer-promoter interactions are initially established at the S0-S1 transition when α-globin expression is activated, and are strengthened in the subsequent S2 and S3 stages, when α-globin expression is further upregulated [9^••^]. This detailed analysis of the α-globin locus and several other erythroid gene loci shows that, in contrast to previous observations [29], enhancer-promoter interactions may not precede upregulation of gene activity, but can form gradually and concomitantly with progressive activation of gene expression. Super-resolution imaging studies have shown that the α-globin cluster is less compact in mature erythroblasts compared to early erythroblasts. This indicates that the increase in interaction frequency between the regulatory elements in the α-globin locus in mature erythroid cells results from dynamic interactions in a decompacted structure, rather than from the formation of a tight, compact structure.

Previous work in cell lines [30] and more recently in primary erythroblasts [31^•^] has shown that the primary function of the enhancers is to increase the recruitment of the pre-initiation complex to the promoters of the α-globin genes. Whether the changes in chromatin structure actively facilitate this process or merely reflect the molecular interactions between enhancers and promoters remains an important open question.

## Conclusions

The α-globin cluster provides a very well-characterized locus to study the dynamic regulation of genome organization and activity during differentiation. In addition to being a valuable experimental model to study these important biological principles, a better understanding of the regulation of the globin genes is also relevant for the clinical management of the thalassemia syndromes [32,33].

A limitation of the current studies of the α-globin and many other gene loci, is that they have predominantly focused on the analysis of populations of cells. Therefore, the precise dynamic mechanisms underlying genome regulation and organization in single cells remain to be discovered. Progress in this direction may address important outstanding questions, including the frequency and distance at which enhancers and promoters interact, the nature of their interactions and the relationship between these interactions and transcription. In addition to established methods to analyze aspects of the dynamic transcriptional (RNA expression) and epigenetic programs (chromatin accessibility, DNA methylation and chromatin structure) in single cells, recent technological advances have enabled the development of several multimodal single-cell omics tools, which allow for the simultaneous profiling of multiple aspects of the transcriptional and epigenetic program in individual cells [34]. Increased application of such tools will lead to a better understanding of the dynamic processes underlying gene regulation during differentiation.

Other important outstanding questions involve the formation of non-membrane bound nuclear compartments in which specific protein and RNA species may be concentrated. It has been suggested that such compartments, which may occupy a separate phase from the surrounding nucleoplasm, have an important function in transcriptional control [24,35]. However, many questions relating to the formation and nature of such compartments and their potential importance for gene regulation remain to be answered. Recent advances in super-resolution and live-cell imaging have the potential to address these questions [36, 37, 38].

We anticipate that integration of single-cell sequencing and imaging approaches will bring answers to these and other outstanding questions in the field in the near future. The application of such approaches to well-characterized gene loci, including the α-globin cluster, will allow for optimal data interpretation to establish the general principles underlying genome organization and gene regulation.

## Conflict of interest statement

Nothing declared.

## References and recommended reading

Papers of particular interest, published within the period of review, have been highlighted as:

• of special interest
•• of outstanding interest

## CRediT authorship contribution statement

**A Marieke Oudelaar:** Writing - original draft. **Robert A Beagrie:** Writing - review & editing. **Mira T Kassouf:** Writing - review & editing. **Douglas R Higgs:** Writing - original draft, Funding acquisition.
